# Association between lumbar endplate damage and bone mineral density in patients with degenerative disc disease

**DOI:** 10.1186/s12891-023-06812-0

**Published:** 2023-09-27

**Authors:** Shan Huang, Ke Lu, Hui-juan Shi, Qin Shi, Ya-qin Gong, Jian-liang Wang, Chong Li

**Affiliations:** 1 Department of Radiology, Affiliated Kunshan Hospital of Jiangsu University, No. 566 East of Qianjin Road, Kunshan, Suzhou, 215300 Jiangsu China; 2 Department of Orthopedics, Affiliated Kunshan Hospital of Jiangsu University, No. 566 East of Qianjin Road, Kunshan, Suzhou, 215300 Jiangsu China; 3 Department of Orthopedics, the First Affiliated Hospital of Soochow University, Orthopedic Institute of Soochow University, Suzhou, 215031 Jiangsu China; 4 Information Department, Affiliated Kunshan Hospital of Jiangsu University, Suzhou, 215300 Jiangsu China

**Keywords:** Total endplate score, Bone mineral density, Osteoporosis, Magnetic resonance imaging, Dual-energy x-ray absorptiometry

## Abstract

**Background:**

To explore the independent association between lumbar endplate damage and bone mineral density (BMD) in patients with degenerative disc disease (DDD).

**Methods:**

This retrospective investigation was based out of a prospectively collected database from the Affiliated Kunshan Hospital of Jiangsu University. Data from 192 DDD patients, collected between December 2018 and January 2022, were chosen for the final analysis. The average total endplate score (TEPS) of lumbar(L) 1-L4 was assessed by magnetic resonance imaging (MRI), and represents the extent of endplate damage. Osteoporosis severity was assessed via the L1-L4 BMD evidenced by dual-energy x-ray absorptiometry (DXA). Other analyzed information included gender, age, body mass index (BMI), and osteophyte score (OSTS). Uni- and multivariate linear regression analyses were employed to evaluate the association between average TEPS and BMD of L1-L4. Moreover, the generalized additive model (GAM) was employed for non-linear association analysis.

**Results:**

Upon gender, age, BMI, and OSTS adjustments, a strong independent inverse relationship was observed between average TEPS and BMD (β, -0.021; 95% CI, -0.035 to -0.007, *P*-value = 0.00449). In addition, the gender stratification analysis revealed a linear relationship in males, and a non-linear relationship in females. Specifically, there was a significantly stronger negative relationship between average TEPS and BMD in females, when the average TEPS was < 3.75 (β, -0.063; 95% CI, -0.114 to -0.013; *P*-value = 0.0157). However, at an average TEPS > 3.75, the relationship did not reach significance (β, 0.007; 95% CI, -0.012 to 0.027; *P*-value = 0.4592).

**Conclusions:**

This study demonstrated the independent negative association between average TEPS and BMD values of L1-L4. Upon gender stratification, a linear relationship was observed in males, and a non-linear association in females. The findings reveal that patients with osteoporosis or endplate damage require more detailed examinations and treatment regimen.

**Supplementary Information:**

The online version contains supplementary material available at 10.1186/s12891-023-06812-0.

## Background

Osteoporosis, an aging-related systemic disease, is marked by reduced bone mass and bone tissue microstructure degeneration. Patients suffering from osteoporosis are prone to fractures, which greatly affect the quality of their lives, and increase mortality [1]. According to the latest nationwide epidemiological osteoporosis survey performed in 2018, osteoporosis prevalence among Chinese postmenopausal women is 32.1%, and among males 50 years or older is 6.9% [2]. Despite its high prevalence, osteoporosis is an undertreated disease. In fact, only 0.3% of males and 1.4% of females with osteoporosis or fracture receive anti-osteoporosis treatment for fracture prevention [2].

Degenerative disc disease (DDD) is another aging-related disease, and the main contributor of lower back pain and disability [3]. Based on the report by Teraguchi M et al. in 2014, > 90% of adults over 50 suffer from DDD [4]. The intervertebral disc is composed of three anatomical structures, namely, the central nucleus pulposus, annulus fibrosus, which circles the nucleus pulposus circumference, and cartilage endplates, which distinguishes the annulus fibrosus and nucleus pulposus from the vertebral bodies [5]. The intervertebral disc is the largest avascular structure within the human body, and it relies on the endplate for nutrition [5]. When the endplate is damaged, its nutritional supply to the disc is reduced, which promotes disc degeneration. The study of Ruiz Wills C et al. indicated that endplate damage is a key factor in DDD [6]. Similarly, Rade M et al. also speculated that vertebral endplate defect promotes DDD [7].

Osteoporosis and DDD are acknowledged common causes of low back pain. Multiple investigations have been carried out to explore the association between osteoporosis and DDD [8–13]. In most prior investigations, the severity of intervertebral disc degeneration has been assessed using the Pfirrmann grading system or a modified version of it [9–13]. These grading systems typically rely on evaluating the degree of degeneration of the nucleus pulposus and annulus fibrosus as the primary scoring criteria. However, the cartilage endplate, an important structure that plays a crucial role in the maintenance of disc homeostasis, is often overlooked and rarely included in the evaluation process. Only a limited number of studies examined the association between osteoporosis and endplate damage. Following certain animal experimentations, some scholars speculated that osteoporosis promotes additional development of endplate damage in animals [14, 15]. In clinical researches, the degree of endplate damage was shown to be an independent contributor to bone mineral density (BMD). Okano I et al. suggested that the endplate damage degree has a strong direct correlation with the regional volume BMD (vBMD), as evidenced by quantitative computed tomography (QCT) [16]. Li R et al. reported that patients with DDD who had higher lumbar BMD tended to have higher rates of endplate damage [17]. In contrast, the study of Zhuang C et al. revealed completely opposite results [18]. A marked inverse relationship was observed between average total endplate score (TEPS) and HU values of L1-L4 in their study, among which the average HU values of L1-L4 represented the BMD. Hence, this investigation aimed to elucidate the potential association between endplate damage degree and BMD, as the clinical evidence was limited and controversial. We hypothesize that there is a potential association between the degree of endplate damage and BMD, and that patients with osteoporosis or endplate damage may require more detailed examinations and treatment regimens.

## Materials and methods

### Study design and patient population

This retrospective investigation is based out of a prospectively collected database (December 2018-January 2022) from our tertiary referral center. For this investigation, 987 consecutive patients, who underwent spinal magnetic resonance imaging (MRI), X-ray examination of the lumbar spine and dual energy x-ray absorptiometry (DXA) examinations were selected for this study. The inclusion criteria were as follows: (1) DDD patients including lumbar intervertebral disc protrusion, degenerative lumbar spondylolisthesis, and degenerative lumbar spinal stenosis [17, 18]. At least one of the aforementioned conditions were present for DDD diagnosis; (2) All MRI and DXA examinations were performed separately on the same machine, and the interval between MRI, X-ray and DXA examinations was < 15 days. The exclusion criteria were as follows: (1) history of lumbar surgery; (2) patients with vertebral fractures; (3) history of anti-osteoporosis or hormone therapy or use of other drugs affecting BMD; (4) patients with BMD-affecting diseases, including, diabetes mellitus, rheumatoid arthritis, leukemia, etc.; (5) missing data or images with severe artifacts. We received ethical approval from the Affiliated Kunshan Hospital of Jiangsu University (approval No. 2022–06-001-K01), and were compliant with the Declaration of Helsinki. Documentation of patient information was initially done to improve hospital care. Owing to the anonymous and observational nature of this study, the written informed consent requirement was waived, and the decision was approved by the Ethics Committee of Kunshan Hospital, Jiangsu University (approval No. 2022–06-001-K01).

### Endplate score

**Fig. 1:**
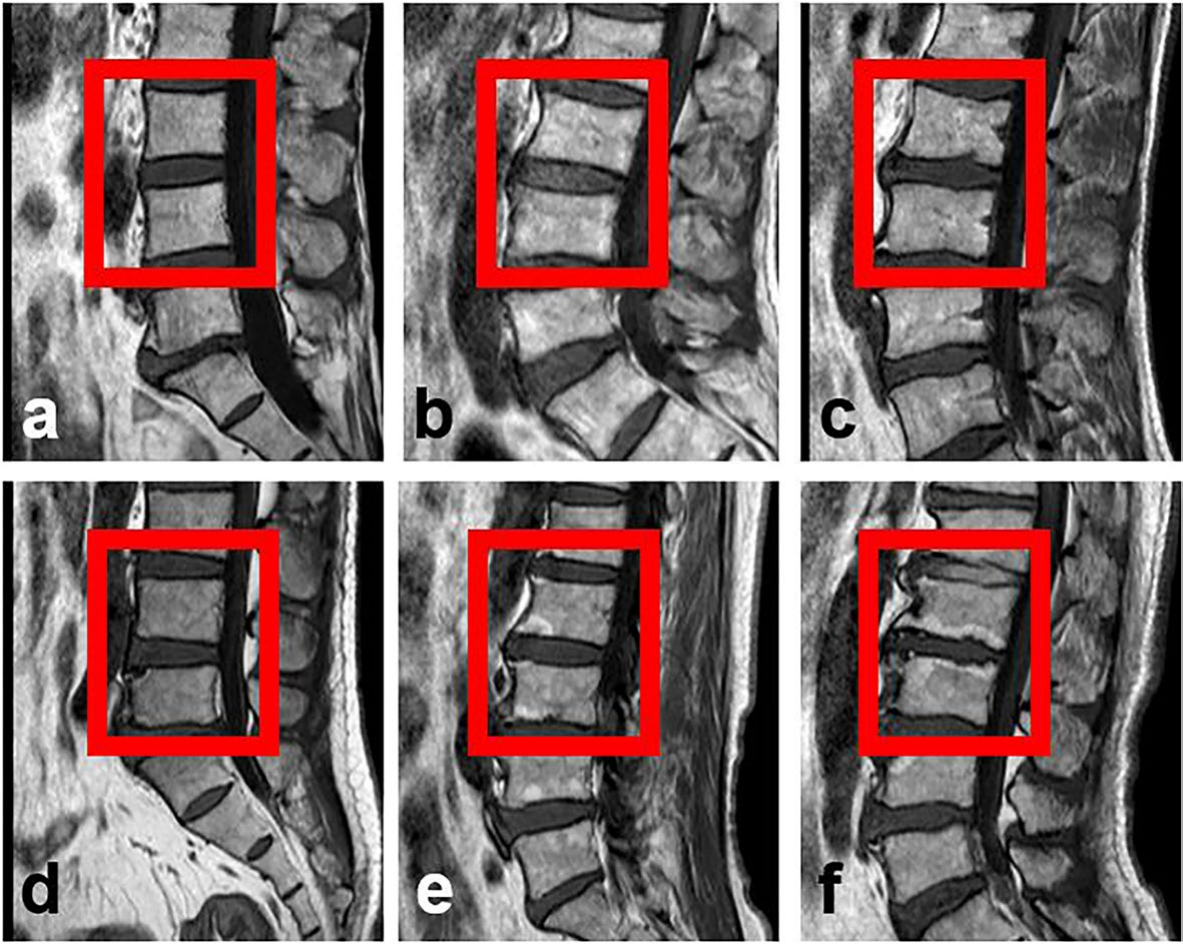
The endplate scoring standard. **a** grade 1: normal endplate with no breaks or defects; **b** grade 2: focal thinning either in the center or periphery, with no endplate breaks or defects; **c** grade 3: focal defects with disc marrow contact; **d** grade 4: defects occupying up to 25% of the entire endplate area; **e** grade 5: breaks occupying up to 50% of the entire endplate area; **f** grade 6: damage to almost the entire endplate area

Every patient underwent 1.5 T MRI scans (Philips Medical Systems, Best, The Netherlands). The endplate damage status was assessed using T1 sagittal images, prior to classification into six categories, based on the method reported by Rajasekaran et al. (Fig. 1) [19]. TEPS was derived from the addition of the cranial and caudal endplate scores of L1/2、L2/3、L3/4 and L4/5 discs. Herein, the average TEPS of L1/2、L2/3、L3/4 and L4/5 discs was used to represent the endplate damage severity of individual patients [18]. The average TEPS were evaluated by two radiologists with 10 and 3 years of experience. Two weeks apart, MRI images of 60 patients were arbitrarily chosen, and physician 1 performed the second evaluation. Both analyzers were blinded to each other and patients’ identifier information.

### BMD assessment

Lumbar DXA scans (Discovery A densitometers, Hologic Inc., Bedford, MA, USA) were used to measure BMD, and the L1-L4 BMD values were recorded to represent the osteoporosis status of patients.

### Osteophyte score

**Fig. 2:**
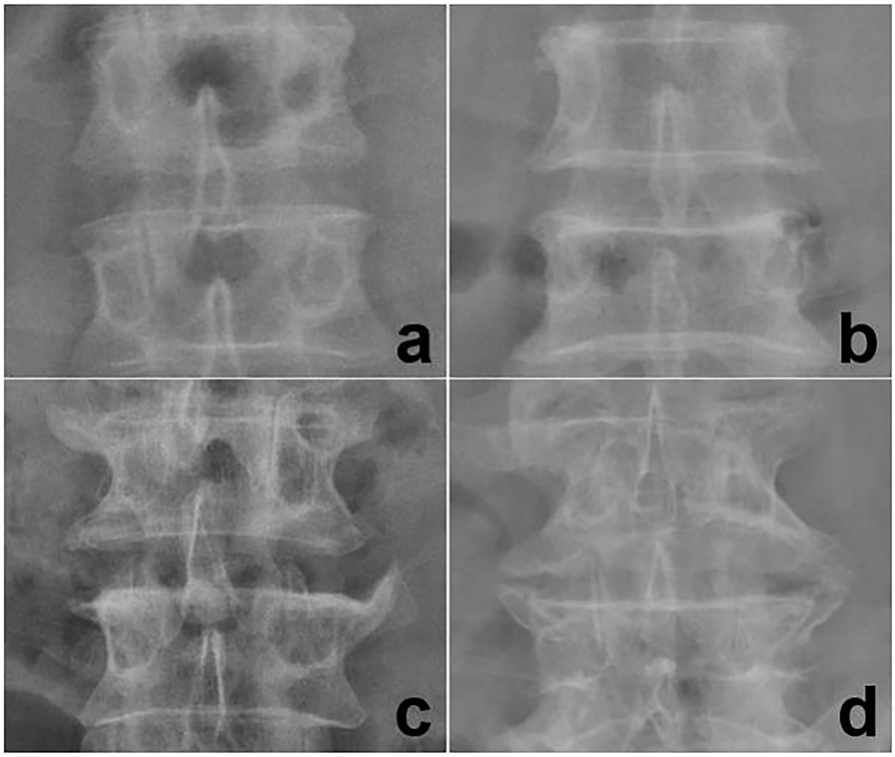
Grading of osteophyte status. **a** grade 1, osteophyte only at the vertebral margins; **b** grade 2, osteophyte almost parallel to the vertebral body, giving the resemblance of a human lip; **c** grade 3, osteophyte with a beak-like appearance, and closer to the adjacent vertebral body; **d** grade 4, fusion of two adjacent vertebral osteophytes

All patients underwent X-ray scans. As DXA overestimate lumbar BMD due to osteophytes [20], osteophyte score (OSTS) was used instead as a covariate to minimize any influence on the association between endplate damage and BMD. The osteophyte degree was graded on a 4-point scale (1 to 4) of the anteroposterior lumbar vertebra on an X-ray image referring to NATHAN's X-ray osteophyte scoring standard (Fig. 2) [21]. The endpoints were coded to establish differences across osteophyte severities, depending on the individual participant’s most severe L1-L4 characteristic [22]. The OSTS were evaluated by two radiologists with 10 and 3 years of experience. Two weeks apart, X-ray images of 60 patients were arbitrarily chosen, and physician 1 conducted the second assessment. Both analyzers were blinded to each other and patients’ identifier information.

### Demographic characteristics

The study collected several indices, including gender, age, and body mass index (BMI).

### Statistics

To explore both inter-observer and intra-observer reliabilities, we conducted a two-way random model, and computed single-measure intraclass correlation coefficients (ICCs) for absolute agreements. The agreement quality assessment used thresholds as follows: ICC values < 0.5: poor reliability; 0.5—0.75: moderate reliability; 0.75—0.9: good reliability, and > 0.90: excellent reliability [23]. Patient demographic and imaging characteristics are presented as mean (standard deviation [SD]) or/and median (first quartile [Q1] to third quartile [Q3]) in case of continuous data, and as frequency (percentage) in case of categorical data. To analyze categorical data, Chi-square or Fisher’s exact test was employed. Normally distributed continuous data was assessed via the independent samples t-test, and non-normally distributed continuous data was assessed via the Mann–Whitney U test.

The study utilized generalized estimating equations (GEE) to analyze the independent association between average TEPS and BMD while accounting for confounding factors. The analysis included unadjusted (crude model), minimally adjusted (Model I), and fully adjusted (Model II) outcomes. Initially, the variance inflation factor (VIF) analysis was performed to conduct diagnoses of covariance collinearities. Next, a covariance decision was made depending on the following criteria: (1) Covariate addition to the basic model or removal from the full model resulted in a corresponding odds ratio (OR) of ≥ 10%; (2) covariate *P*-value of < 0.1, based on univariate analysis or criteria (1) [24].

Non-linear associations were screened for using the generalized additive model (GAM), and the threshold was computed based on the smoothing curve using a two-piecewise linear regression model. Once a clear ratio was obtained, the recursive method was used to automatically generate the turning point (K) for the maximum likelihood model [25]. Moreover, to examine the strength and possible heterogeneity in the distinct subgroup, subgroup analyses was conducted alongside the simultaneous stratification of varying covariates. The subgroup modifications and interactions were evaluated via the likelihood ratio test (LRT).

The SPSS Statistics version 22.0 package (IBM Corp., Armonk, NY, USA), Empower Stats (www.empowerstats.com, X&Y solutions, Inc., Boston, MA, USA), and R version 3.6.3 (http://www.r-project.org) were employed for data analyses. *P*-value < 0.05 was deemed as significant.

### Sample size

According to the EPV (events per variable) principle, 192 patients in this study is sufficient [26].

## Results

### ICC analysis results

The median and interquartile spacing of the average TEPS for physician 1 was 4.50 (3.25—5.75), and for physician 2 was 5.00 (4.00—6.50). The inter-observer reliability for the average TEPS was good for both physicians (ICC, 0.879; 95% CI, 0.671—0.941; *P*-value < 0.001). The intra-observer reliability for the average TEPS was also good (ICC, 0.883; 95% CI, 0.809—0.929; *P*-value < 0.001). The results of physician 1 were as follows: 55, 50, 61, 26 patients with OSTS 1, 2, 3, 4, respectively, and that of physician 2 were as follows: 54, 57, 56, 25 patients with OSTS 1, 2, 3, 4, respectively. The inter-observer OSTS reliability was good for both physicians (ICC, 0.830; 95% CI, 0.780—0.869; *P*-value < 0.001). The intra-observer reliability for OSTS was also good (ICC, 0.869; 95% CI, 0.791—0.920; *P*-value < 0.001). In subsequent evaluations, the results from the senior physician (physician 1) were utilized.

### Patient profile and univariate analysis

**Fig. 3:**
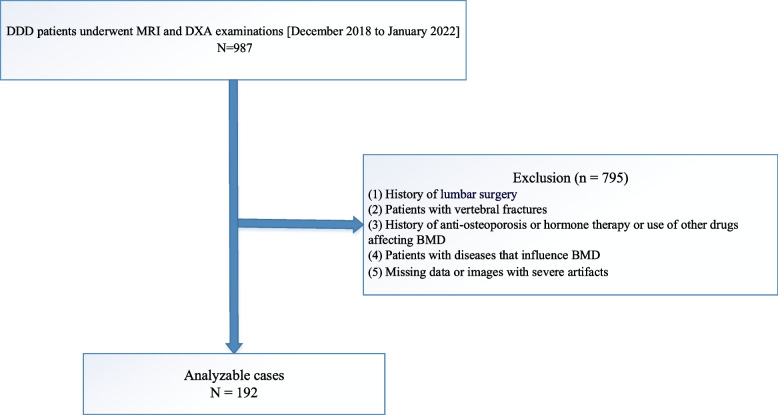
A schematic diagram of the study design. DDD, degenerative disc disease; MRI, magnetic resonance imaging; DXA, dual-energy x-ray absorptiometry; BMD, bone mineral density

Out of the initial 987 patients, 795 were excluded, and the remaining 192 patient records aged between 30 and 87 years were selected for analysis. Among them, female patients accounted for 53.65%. A schematic diagram of the patient selection process is presented in Fig. 3. Table 1 depicts patient demographics and presents the outcomes of the univariate analysis in terms of average TEPS, gender, age, OSTS and BMD univariate analysis. Based on the univariate analysis, there was a significant negative correlation observed between average TEPS and BMD, indicating that for each one-unit increase in average TEPS, BMD decreased by 0.021 g/cm^2^ (*P*-value = 0.00229). Secondly, women exhibited a 13.3% lower BMD, compared to men (*P*-value < 0.00001). Thirdly, BMD was reduced by 0.004 g/cm^2^ for each 1-year rise in age (*P*-value = 0.00008). Fourthly, a one-unit rise in BMI corresponded to a 0.014 g/cm^2^ elevation in BMD (*P*-value = 0.00040). Finally, at OSTS of 2, BMD diminished by 0.102 g/cm^2^ relative to OSTS 1 (*P*-value = 0.00230). However, no significance was reached when OSTS were 3 and 4 (both *P*-value > 0.05).

**Table 1:** Patient profile and univariate analysis

Variables	(N) Mean (SD) Median (Q1-Q3) / N (%)	β^a^ (95% CI) *P*-value
Average TEPS	(192) 4.70 (1.86) 4.50 (3.25–5.75)	-0.021 (-0.034, -0.008) 0.00229
Gender, N (%)		
Male	89(46.35%)	Reference
Female	103(53.65%)	-0.133 (-0.179, -0.087) < 0.00001
Age, y	(192) 60.41(12.28) 63.00(51.00–70.00)	-0.004 (-0.006, -0.002) 0.00008
BMI, kg/m^2^	(192) 24.43(3.19) 24.22(22.65–26.31)	0.014 (0.006, 0.021) 0.00040
OSTS		
1	55(28.65%)	Reference
2	50(26.04%)	-0.102 (-0.167, -0.037) 0.00230
3	61(31.77%)	-0.053 (-0.114, 0.009) 0.09376
4	26(13.54%)	0.057 (-0.022, 0.136) 0.15627

*Abbreviations*: *SD* Standard deviation, *Q1* First quartile, *Q3* Third quartile, *TEPS* Total endplate score, *BMD* Bone mineral density, *BMI* Body mass index, *OSTS* Osteophyte score, *CI* Confidence interval

^a^The dependent variable was BMD of L1-L4

### The independent relationship between the average TEPS and BMD

Table 2 summarizes the independent relationship between the average TEPS and BMD, as evidenced by multivariate analysis. A two-level adjustment was employed based on covariance analysis: the crude model was not adjusted; Model I was adjusted for gender, age, and OSTS; Model II was adjusted for Model I plus BMI. A strong inverse relationship existed between average TEPS and BMD in the crude model (β, -0.021; 95% CI, -0.034 to -0.008; *P*-value = 0.00229), Model I (β, -0.021; 95% CI, -0.035 to -0.006; *P*-value = 0.00724) and Model II (β, -0.021; 95% CI, -0.035 to -0.007; *P*-value = 0.00449). These results can be further interpreted as follows: A one-unit rise in average TEPS correlated with a 0.021 g/cm^2^ reduction in the BMD in the crude model, Model I, and Model II.

**Table 2:** Relationship between average TEPS and BMD in different models

	β	95% CI	*P*-value
Crude Model^a^	-0.021	(-0.034, -0.008)	0.00229
Model I^b^	-0.021	(-0.035, -0.006)	0.00724
Model II^c^	-0.021	(-0.035, -0.007)	0.00449

*Abbreviations*: *CI* Confidence interval, *TEPS* Total endplate score, *BMD* Bone mineral density, *BMI* Body mass index, *OSTS* Osteophyte score

^a^No adjustment

^b^Adjusted for gender, age, OSTS

^c^Adjusted for Model I plus BMI

### The threshold analysis and spline smoothing plot

**Fig. 4:**
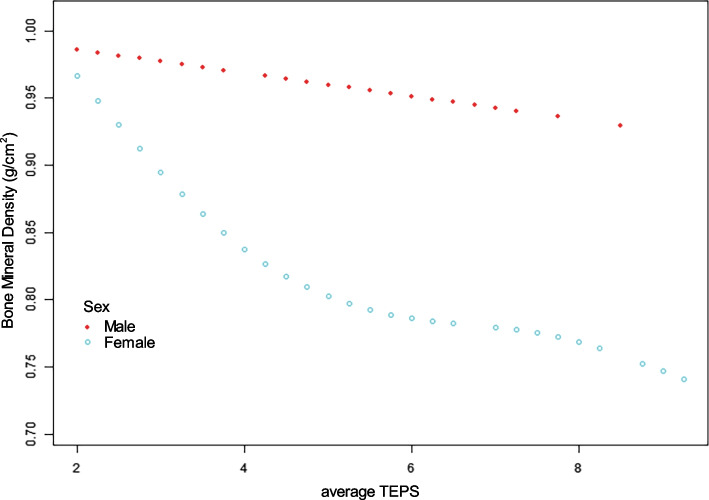
The adjusted smoothed curves of average TEPS and BMD of L1-L4 stratified by gender. The red line denotes the smooth curve of males. The blue line denotes the smooth curve of females. A linear association between average TEPS and BMD for males, and a non-linear association for females, based on the generalized additive model. The adjustment factors were age, BMI and OSTS. CI, confidence interval; TEPS, total endplate score; BMD, bone mineral density; BMI, body mass index; OSTS, osteophyte score

Table 3 lists the threshold effect analysis, which examined the relationship between average TEPS and BMD in the fully adjusted Model II, based on sex stratification. An LRT* P*-value < 0.05 indicated a non-linear association. Analysis revealed a non-linear association between average TEPS and BMD in females. The two-piecewise linear regression model was used to compute the turning point (K) of the adjusted smoothed curve, which was found to be 3.75 of the average TEPS for females. A significantly stronger negative relationship between average TEPS and BMD was observed when the average TEPS was below 3.75 (β, -0.063; 95% CI, -0.114 to -0.013; P-value = 0.0157). However, at an average TEPS above 3.75, the association did not reach significance (β, 0.007; 95% CI, -0.012 to 0.027; *P*-value = 0.4592). In contrast, a linear relationship existed between average TEPS and BMD in males (β, -0.028; 95% CI, -0.051 to -0.004; P-value = 0.0218). The adjusted spline smoothing plot visually illustrates these results (Fig. 4). Based on the threshold effect analysis and adjusted spline smoothing plot results, a more obvious negative association was observed in female patients, compared to male patients.

**Table 3:** Threshold effect analysis examining the relationship between average TEPS and BMD^a^

	Male	Female	β (95% CI) *P*-value
Model A^b^			*P* for interaction: 0.0852
One line slope	-0.028 (-0.051, -0.004) 0.0218	-0.006 (-0.022, 0.010) 0.4735	-0.021 (-0.035, -0.007) 0.0045
Model B^c^			*P* for interaction: 0.034
Turning point (K), Average TEPS	2.5	3.75	5
< K	0.133 (-0.093, 0.359) 0.2520	-0.063 (-0.114, -0.013) 0.0157	-0.034 (-0.060, -0.009) 0.0091
> K	-0.035 (-0.059, -0.010) 0.0082	0.007 (-0.012, 0.027) 0.4592	-0.009 (-0.033, 0.014) 0.4415
Slope 2—Slope 1	-0.167 (-0.402, 0.067) 0.1650	0.071 (0.012, 0.130) 0.0209	0.025 (-0.015, 0.065) 0.2202
LRT^d^	0.144	**0.016**	0.209

*Abbreviations*: *CI* Confidence interval, *TEPS* Total endplate score, *BMD* Bone mineral density, *BMI* Body mass index, *OSTS* Osteophyte score, *LRT* Logarithmic likelihood ratio test

^a^Adjusted for gender, age, OSTS and BMI in Model II

^b^Linear analysis, *P*-value < 0.05 indicates a linear relationship

^c^Non-linear analysis

^d^*P*-value < 0.05 means Model B is significantly different from Model A, which indicates a non-linear relationship

### Subgroup analysis

To further confirm that the findings were robust to potential confounders in the fully adjusted Model II, subgroup analyses were performed while stratifying by gender, age, BMI, and OSTS. All analyses were adjusted for the aforementioned four covariates, except for the subgroup variable. Table 4 summarizes a highly consistent pattern, and no interactions were observed based on all stratifications (all *P*-values for interaction > 0.05), except gender. Gender exhibited no statistically significant interaction in the linear model (*P*-value for interaction = 0.0852), but a strong interaction in the non-linear model (*P*-value for interaction = 0.034) (Table 3).

**Table 4:** Subgroup analyses examining the relationship between average TEPS and BMD

Subgroup	N	β (95% CI) *P*-value	*P*-value for interaction
Gender			0.034^a^
Male	89	-0.028 (-0.051, -0.004) 0.0218	
Female	103	-0.006 (-0.022, 0.010) 0.4735	
Age tertile			0.9608
Tertile 1 (30—54 y)	61	-0.000 (-0.025, 0.024) 0.9829	
Tertile 2 (55—67 y)	67	-0.019 (-0.043, 0.006) 0.1362	
Tertile 3 (68—87 y)	64	-0.016 (-0.039, 0.006) 0.1485	
BMI tertile			0.5856
Tertile 1 (17.78—23.18) kg/m^2^)	64	-0.021 (-0.047, 0.005) 0.1170	
Tertile 2 (23.19—25.61 kg/m^2^)	63	-0.028 (-0.054, -0.001) 0.0463	
Tertile 3 (25.65—37.18 kg/m^2^)	65	-0.007 (-0.033, 0.018) 0.5626	
OSTS			0.2486
1	55	-0.005 (-0.043, 0.034) 0.8134	
2	50	-0.023 (-0.046, 0.000) 0.0600	
3	61	-0.021 (-0.046, 0.003) 0.0955	
4	26	-0.001 (-0.028, 0.026) 0.9477	

Adjusted for gender, age, BMI, OSTS except the subgroup variable

*Abbreviations*: *CI* Confidence interval, *TEPS* Total endplate score, *BMD* Bone mineral density, *BMI* Body mass index, *OSTS* Osteophyte score

^a^For non-liner model

## Discussion

This investigation confirmed an independent association between average TEPS and BMD of L1-L4 in DDD patients. Using different adjusted models, the negative association remained stable, which was consistent with the study by Zhuang C et al. [18]. A potential mechanism behind the osteoporosis and endplate damage association is yet unknown. Huang B et al. suggested that inflammation is linked to endplate abnormalities, as they found significantly elevated levels of tumor necrosis factor-α (TNF-α) in the micro-damaged endplate compared to control specimens in their study [27]. Osteoporosis is also linked to inflammatory factors, as ageing and estrogen deficiency can activate the immune system at a low level and trigger a pro-inflammatory response. Inflammatory factors such as TNF-α, interleukin-1 (IL-1), IL-17 can stimulate the production of macrophage colony-stimulating factor (M-CSF) and receptor activator of nuclear factor-kB (NF-kB) ligand (RANKL), which promote osteoclast formation and inhibit osteoblasts, thereby, resulting in reduced bone mass [28]. This mechanism could potentially explain the association between osteoporosis and endplate damage.

The gender-stratified analysis revealed a gender interaction in the relationship between average TEPS and BMD, suggesting the need for separate analysis of the relationship for males and females (*P*-value for interaction = 0.034). The threshold effect analysis further revealed that the *P*-value for interaction in linear model was slightly > 0.05 (*P*-value for interaction = 0.0852), but the *P*-value for interaction in non-linear model was < 0.05 (*P*-value for interaction = 0.034). These results suggested that the significant gender interaction was based on the non-linear model. Upon stratification by gender, there was a linear negative relationship between average TEPS and BMD in males, such that every unit rise in average TEPS was associated with a decrease of 0.028 g/cm^2^ in BMD. In females, a non-linear negative association was observed, whereby, the association in terms of values of average TEPS greater or less than the K value (K = 3.75 is the inflection point in the non-linear relationship) was inverse of each other. The average TEPS was strongly and inversely proportional to BMD on the left side of inflection, but showed no significance on the right side of inflection. This may explain the divergence of the studies by Zhuang C et al. and Okano I et al. [16, 18]. The majority of patients in the study of Okano I et al. were females (58.3%), and the positive relationship between TEPS and BMD only appeared in the elevated TEPS groups (TEPS 10–12) [16]. In addition, based on the threshold effect analysis and adjusted spline smoothing plot analysis, female patients displayed a stronger inverse association between average TEPS and BMD compared to male patients. The associated mechanisms warrant further exploration.

This study holds clinical significance as it confirms the independent negative correlation between average TEPS and BMD of L1-L4. MRI can provide additional information on the degree of osteoporosis while assessing endplate damage, making it a potentially supplementary tool to DXA, particularly in areas where DXA equipment is limited or in patients with severe osteophyte. Furthermore, the negative correlation between average TEPS and BMD implies that severe endplate damage is associated with lower BMD. This suggests that endplate damage, in addition to osteoporosis, may contribute to low back pain in patients with osteoporosis, requiring further MRI examination, while those with severe endplate damage may benefit from additional DXA examination for osteoporosis. Moreover, gender-stratified analysis showed that female patients with elevated average TEPS are at a higher risk for diminished BMD. Therefore, additional care, such as drug combination therapy, specific exercise modality, etc. are required in the clinical management of female patients with osteoporosis or DDD [29–32].

This investigation has several strengths. Firstly, unlike the use of CT values in other investigations, DXA-measured BMD was utilized in this study, and OSTS was adopted as a covariate to control its influence on the relationship of average TEPS and BMD. Secondly, the generalized linear model was employed to assess the linear association between average TEPS and BMD, and simultaneously used GAM to elucidate the non-linear association. GAM is known to be beneficial for the analysis of non-linear interactions, and can perform non-parametric smoothing to fit a regression spline to the data. Hence, we used this technology to better elucidate the true relationship between average TEPS and BMD. Furthermore, previous studies have often overlooked the influence of gender on endplate damage and BMD [16, 18]. Herein, gender stratification analysis was employed in this study to reveal gender differences in the association between average TEPS and BMD, which is indicative of a need for distinct clinical treatment regimens for male and female patients.

This study has some limitations. The first is the limited sample size. Secondly, the average TEPS and OSTS evaluations were entirely radiological and subjectively estimated. Even though highly skilled physicians collected and computed the data, measurement bias may still exist. Moreover, this study was an analytical retrospective investigation and, thus, any associations observed here will not represent causation. Thus, the causal relationship between endplate damage and osteoporosis requires further investigation, for example, multicenter randomized controlled trials (RCTs) or long time cohort studies. Furthermore, due to the retrospective design of this study, we were unable to retrieve information about possible medications that was not clinically recorded, which may have been taken by the selected patients.

## Conclusions

To sum up, this study demonstrated the independent negative association between average TEPS and BMD of L1-L4. Upon gender stratification, a linear relationship was further revealed in males, whereas a non-linear relationship was found in females. These findings hold certain value for the clinical examination and therapy of patients with osteoporosis or endplate damage.

### Supplementary Information

**Additional file 1.**

## Data Availability

The dataset supporting the conclusions of this article is included within the article and its additional file.

## Abbreviations

BMD: Bone mineral density
DDD: Degenerative disc disease
TEPS: Total endplate score
L: Lumbar
MRI: Magnetic resonance imaging
DXA: Dual-energy X-ray absorptiometry
BMI: Body mass index
OSTS: Osteophyte score
QCT: Quantitative computed tomography
ICCs: Intraclass correlation coefficients
